# Trajectory of change in perceived stress, coping strategies and clinical competence among undergraduate nursing students during clinical practicum: a longitudinal cohort study

**DOI:** 10.1186/s12909-024-05332-2

**Published:** 2024-03-29

**Authors:** Li-Hung Tsai, Lai-Chu See, Jun-Yu Fan, Ching-Ching Tsai, Chuan-Mei Chen, Wei-Sheng Peng

**Affiliations:** 1 Department of Nursing, Chang Gung University of Science and Technology, 261, Wenhua 1St Rd., Guishan Dist., Taoyuan City, 33303 Taiwan; 2 Biostatistics Core Laboratory, Molecular Medicine Research Center, Chang Gung University, Taoyuan City, Taiwan; 3 Department of Public Health, College of Medicine, Chang Gung University, Taoyuan City, Taiwan; 4 Division of Rheumatology, Allergy and Immunology, Department of Internal Medicine, Chang Gung Memorial Hospital, Linkou, Taiwan; 5 Department of Nursing & Graduate Institute of Nursing, Chang Gung University of Science and Technology, Taoyuan City, Taiwan; 6 Division of Nursing, Chang Gung Memorial Hospital, Linkou, Taiwan; 7 Department of Cardiology, Chang Gung Memorial Hospital, Linkou, Taiwan; 8 Department of Nursing, Central Taiwan University of Science and Technology, Taichung City, Taiwan

**Keywords:** Undergraduate nursing student, Clinical practicum, Perceived stress, Coping strategy, Clinical competence

## Abstract

**Background:**

Clinical practicum is crucial for strengthening nursing students' clinical competence. However, nursing students often experience considerable stress during clinical practicum, and so they employ coping strategies to alleviate it. There is almost no empirical evidence on the change trajectory of perceived stress, coping strategies, and clinical competence among nursing students during a one-year clinical practicum. This study aimed to investigate the trajectory of change in perceived stress, coping strategies, and clinical competence among undergraduate nursing students during a one-year clinical practicum.

**Methods:**

This study used a longitudinal cohort design. Undergraduate nursing students were recruited from a science and technology university in Taiwan to participate from February 2021 to January 2022. Perceived stress, coping strategies, and clinical competence among students in basic training practicum (T1), advanced training practicum (T2), and comprehensive clinical nursing practicum (T3) were surveyed by using the Perceived Stress Scale (PSS), Coping Behaviour Inventory (CBI), and Clinical Competence Scale (CCS). PSS, CBI, and CCS in T1, T2, and T3 were compared using a generalized estimating equation (GEE) to deal with correlated data. The level of statistical significance was set at α = 0.05.

**Results:**

A total of 315 undergraduate nursing students completed the questionnaire. The study results show that the overall perceived stress of the students is the highest in T2 and the lowest in T3. The main source of stress of the students is 'taking care of patients' at T1 and 'lack of professional knowledge and skills' at T2 and T3. Students' perceived stress in 'taking care of patients' gradually decreases over time. The four coping strategies of CBI, which are 'stay optimistic', 'problem-solving', 'transference' and 'avoidance' in this order, remain the same ranking in three surveys.The main stress coping strategy used by students is 'stay optimistic', while the coping strategy 'avoidance' is used more frequently in T2 than in T1 and T3. Students' mean scores of the overall clinical competence and in the 'general nursing' and 'management' subscales in T3 are higher than those in T1 and T2. However, their mean scores in 'self-growth' and 'positivity' subscales are the highest in T1 and the lowest in T2.

**Conclusions:**

The results show that through experiential learning in clinical practicum at different stages time after time, students' overall perceived stress is the lowest and their overall clinical competence is the highest in T3. The main coping strategy used when students managed stress is 'stay optimistic'. According to the results, we suggest that clinical educators provide students with appropriate guidance strategies at different stages of stress and continue to follow up the clinical competence and retention rates of these nursing students in the workplace in the future.

## Introduction

The mission of all nursing programmes is to educate students to become competent nurses. Upon graduation, nursing students are expected to have sufficient high-level competence to provide effective and high-quality nursing care [1]. A clinical practicum course serves a crucial role in strengthening the clinical competence of nursing students. In addition, the experiences during clinical practicum had a significant effect on future career and workplace choices [2]. However, during practicums, nursing students encounter various difficulties and challenges. Recent studies highlighted that a significant percentage of nursing students, ranging from 63.5% to 81.2%, experience moderate to high-stress levels during their clinical practice [3, 4]. Students' stress in their clinical practice can be altered and influenced by the coping strategies they choose to employ [5]. Ineffective coping can negatively affect the self-concept, learning skills and competence of students [6]. Clinical practicum is essentially experiential learning, and experiential learning is learning achieved through the appropriate use of experience. Therefore, nursing schools arrange a series of clinical practicum courses for students so that students can accumulate professional knowledge and skills through repeated experiential learnings and become competent nurses after graduation. Ferro Allodola [7] conducted a literature review and found that experiential learning is effective in enhancing the clinical and practice-based skills and knowledge of students. Similarly, can nursing students lower the intensity of perceived stress with experience accumulation through experiential learning in clinical practicum at different stages time after time? Moreover, how do students' coping strategies and clinical competence change with time during the clinical practicum? These are topics worth paying attention to as they can serve as references for clinical teaching plans.

## Background

A competent nursing staff should have some clinical competence, such as general nursing, cooperation, management, self-growth, stress adjustment, innovation and research [8]. Nursing schools typically rely on a clinical agency practicum to support students in the simulation of didactic theory to application [9]. A clinical practicum is essentially experiential learning, which is a form of learning by doing. The experiential learning model of Kolb is a continuous spiral process consisting of four basic elements: concrete experience, observation and reflection, forming abstract concepts and testing in new situations. Concrete experiences are the basis for observation and reflections. These reflections are assimilated and distilled into abstract concepts from which new implications for action can be drawn [7, 10]. Therefore, nursing students are expected to become competent nurses through a series of clinical practicums arranged by colleges at different stages and experiential learning at different levels.

During clinical practicum, many situations may cause heavy stress on nursing students, such as (1) entering a new environment, (2) lack of professional competence, (3) fear of making a mistake, (4) fear of harming patients, (5) worry hampers establishing therapeutic relationships with patients, (6) traumatic experiences, (7) anxiety felt about instructor–student interactions or (8) knowing that they are being evaluated by instructors [11–15]. In general, a low to moderate level of stress can motivate students' learning. However, a higher level of stress can affect their physical and mental health and their ability to study [16–18].

The differences in their grades and level of clinical experience will affect nursing students' stress levels. The study has shown that first-year nursing students have the highest level of stress in clinical practice, where the higher the grade, the lower the level of stress [17]. However, another study has shown that first-year nursing students have the least stress levels in clinical practice, where the higher the grade, the higher the level of stress [3]. Gurková and Zeleníková [16] reported that experienced nursing students perceive higher levels of stress than novice students do. In longitudinal cohort studies, Mazalová et al. [19] also reported that the highest degree of stress was recorded at the beginning of a student's studies, after which the stress decreased. In the third year, when students were required to work due to the COVID pandemic, the stress reached the first-year levels again. Zupiria Gorostidi et al. [20] found that compared with first-year students, second- and third-year students tend to perceive lower levels of stress. By contrast, Bhurtun et al. [21] reported that students perceive more stress in their second clinical practice compared to the first one.

In general, the coping strategies used by nursing students vary according to the level and source of their stress [22, 23]. The two types of coping strategies are problem-based and emotion-based. Problem-focused coping strategy is directed towards reducing the stress by targeting the root causes of the stress. Emotion-focused coping strategy is intended to lessen, control and manage adverse emotional responses. Behaviours under this coping method include avoidance and utilisation of self-distraction activities, such as watching TV, snacking and sleeping [5, 21, 23].

Recently, most studies have been designed to explore the perceived stress and coping strategies of nursing students in clinical practicum through a cross-sectional [4, 5, 24] or longitudinal follow-up for two or three years [19, 21]. At present, there is no related study on the long-term tracking of changes in nursing students' clinical competence during practicum. Taiwan's Ministry of Examination clearly stipulates that a nurse must complete a minimum of 1,016 practicum hours as a requirement for the Nurse License Examination. The practicum sections include fundamental nursing, medical and surgical nursing, maternity nursing, paediatric nursing, community health nursing, psychiatric nursing and comprehensive clinical nursing [25]. All four-year bachelor's nursing students in our college should complete their required nursing courses before they take one-year practicum courses. The practicum course is planned as follows: basic training practicum, advanced training practicum, and comprehensive clinical nursing practicum. Therefore, we wanted to investigate the trajectory of change in perceived stress, coping strategies and clinical competence among undergraduate nursing students during their one-year practicum.

## Methods

### Aim

This study investigates the trajectory of change in perceived stress, coping strategies, and clinical competence of four-year bachelor's nursing students in a science and technology university in Taiwan during their one-year clinical practicum.

The following research questions guided this study:

1. What trajectory of change occur in the perceived stress levels of nursing students when comparing their basic training practicum, advanced training practicum and comprehensive clinical practicum?
2. What trajectory of change occur in the coping strategies of nursing students when comparing their basic training practicum, advanced training practicum and comprehensive clinical practicum?
3. What trajectory of change occur in the clinical competence of nursing students when comparing their basic training practicum, advanced training practicum and comprehensive clinical practicum?

### Study design and participants

This study used a longitudinal cohort design. The Institutional Review Board of the concerned hospital approved this study (201901913B0). Purposeful sampling is adopted, and students at Grade 3 in a science and technology university for a four-year bachelor's nursing programme in the north of Taiwan are taken as the object of study. A total of 489 students are participating in the practicum course. Among them, 436 students agree to participate and sign a consent letter after the study is explained before their practicum. Students' practicum was from February 2021 to January 2022 and was divided into three stages: basic training practicum (T1) (from February 2021 to June 2021), advanced training practicum (T2) (from April 2021 to November 2021) and comprehensive clinical nursing practicum (T3) (from September 2021 to January 2022). At the end of each practicum stage, students are requested to fill out questionnaires. At the end of T1, T2, and T3, a total of 397, 397, and 315 students complete valid questionnaires, respectively. Among the 82 students, 40 didn't finish valid questionnaires, and 42 refused to fill in the questionnaire.Therefore, this study is based on the data of 315 students. In addition, no statistical difference is found in the demographic data between students who completed valid questionnaires three times (315) and students who failed to complete valid questionnaires (82) (Table 1).

**Table 1:** Demographic characteristics of students who completed and did not complete the three T1-T3 questionnaires

Item	Total (*n* = 397)	complete (*n* = 315)	non-complete (*n* = 82)	chi-square	*p*-value
**Gender**					
Male	51 (12.85%)	39 (12.38%)	12 (14.63%)	0.30	0.5870
Female	346 (87.15%)	276 (87.62%)	70 (85.37%)		
**Age**	20.40 ± 0.65	20.33 ± 0.50	20.42 ± 0.68	-1.34	0.1831
**Student clubs participation**					
No	128 (32.24%)	104 (33.02%)	24 (29.27%)	0.42	0.5178
Yes	269 (67.76%)	211 (66.98%)	58 (70.73%)		
**Non-nursing part-time job**					
No	138 (34.76%)	115 (36.51%)	23 (28.05%)	2.05	0.1519
Yes	259 (65.24%)	200 (63.49%)	59 (71.95%)		
**Ever-smokers**					
No	391 (98.49%)	309 (98.1%)	82 (100%)	1.59	0.2079
Yes	6 (1.51%)	6 (1.9%)	0 (0%)		
**Ever-drinkers**					
No	353 (88.92%)	278 (88.25%)	75 (91.46%)	0.68	0.4096
Yes	44 (11.08%)	37 (11.75%)	7 (8.54%)		

### Clinical practicum

In our college, four-year bachelor's nursing students are required to complete the clinical practicum course in one year from the second semester of Grade 3 to the first semester of Grade 4. Students must complete fundamental nursing practicum (96 h) and medical-surgical nursing practicum I (135 h) in T1; medical-surgical nursing practicum II (135 h), maternity nursing practicum (135 h), paediatric nursing practicum (135 h), psychiatric nursing practicum (135 h) and community health nursing practicum (135 h) in T2; and comprehensive clinical nursing practicum (216 h) in T3. According to the number of hospital units and the staff allocation of the clinical nursing teachers, students are arranged to complete T1 first, then T2 (students complete five stages of practicum in different order), and finally T3. In T1 and T2, our college assign clinical nursing teachers to guide students in clinical practice. In T3, the hospital's preceptor is responsible for guiding students in clinical practice. Students in this study are arranged to complete nursing practicum courses from February 2021 to January 2022.

### Instruments

#### Demographic data

The demographic data sheet includes gender, age, student clubs participation, non-nursing part-time job experience, ever-smokers and ever-drinkers.

#### Perceived stress scale (PSS)

The PSS, which was developed by Sheu et al. [26], is a five-point Likert scale that examines stress and stressors among nursing students. Responses to each item range from '*never*' to '*always*' (4 = *always*, 3 = *frequently*, 2 = *sometimes*, 1 = *infrequently* and 0 = *never*). The scale comprises 29 items divided into six subscales: eight related to taking care of patients, six related to teachers and nursing staff, five related to assignments and workload, four related to peers and daily life, three related to lack of professional knowledge and skills and three related to the clinical environment. Mean scores of each item of subscale and overall scale were calculated, with higher scores indicating higher levels of stress. Cronbach's alpha of the PSS has been reported to be 0.89 [26]. In this study, Cronbach's alpha for T1, T2, and T3 were 0.93, 0.92, 0.93 respectively.

#### Coping behaviour inventory (CBI)

The CBI, which was developed by Sheu et al. [27], is a five-point Likert scale used to identify the coping strategies of nursing students. Responses to each item range from '*never*' to '*always*' (4 = *always*, 3 = *frequently*, 2 = *sometimes*, 1 = *infrequently* and 0 = *never*). The scale comprises 19 items divided into four subscales: six for avoidance behaviours, six for problem-solving behaviours, four for stay optimistic behaviours and three for transference behaviours. Mean scores of each item of subscale were calculated, with higher scores indicating a higher frequency of utilization. Cronbach's alpha of the CBI has been reported to be 0.76 [27]. In this study, the Cronbach's alpha for T1, T2, and T3 respectively were 0.82, 0.82, 0.83 for avoidance, 0.82, 0.81, 0.81 for problem-solving, 0.80, 0.79, 0.81 for stay optimistic, and 0.70, 0.63, 0.57 for transference. Cronbach's alpha from 0.50 to 0.75 suggests moderate reliability, and above 0.75 indicates good reliability [28].

#### Clinical competence scale (CCS)

After referring to the literature and comprehensive clinical experience, the investigator created a questionnaire, through which the students self-assessed their clinical competencies. The five-point Likert scale (1–5 points) was adopted. Responses to each item range from '*extremely disagree*' to '*extremely agree*' (5 = *extremely agree*, 4 = *agree*, 3 = *neutral*, 2 = *disagree* and 1 = *extremely disagree*). The scale comprises 45 items divided into five subscales: twenty-seven for general nursing, five for cooperation, four for management, three for self-growth and six for positivity (including stress adjustment, sense of responsibility and service enthusiasm). Mean scores of each item of subscale and overall scale were calculated, with higher scores indicating better clinical competence. Six nursing teachers and senior nursing staff were invited to test the expert validity of the scale. These experts were asked to score the questionnaire according to its universality, availability, definition and appropriateness. The result showed that CVI was 0.97–1.0. In this study, Cronbach's alpha for T1, T2, and T3 were 0.95, 0.96, 0.96 respectively.

### Data analysis

Data were analysed using SAS 9.4 statistical software. The chi-square test was made to compare the data between those who completed and those who did not complete the three T1-T3 questionnaires. PSS, CBI, and CCS in T1, T2, and T3 were compared using a generalized estimating equation (GEE) to deal with correlated data. Unlike repeated measure analysis of variance (RMANOVA), GEE does not require the homogeneity of variance. The within-subject correlation matrix in GEE was exchangeable (the correlation between any two response variables at different time points is the same). Multiple comparisons in GEE were made to locate the difference for T1, T2, T3 using the contrast, a set of weights that defines a specific comparison over means. The level of statistical significance was set at α = 0.05.

## Results

### Demographic characteristics

Among 315 students who completed questionnaires of T1-T3, 39 (12.38%) were male. The mean age of the students was 20 years old. Two-thirds of the students joined student clubs. More than a half had an experience in non-nursing part-time jobs (63.49%). Only a few ever-smokers (1.9%), and 11.75% ever-drinkers (Table 1).

### Trajectory of change in perceived stress of nursing students during their practicum

**Fig. 1:**
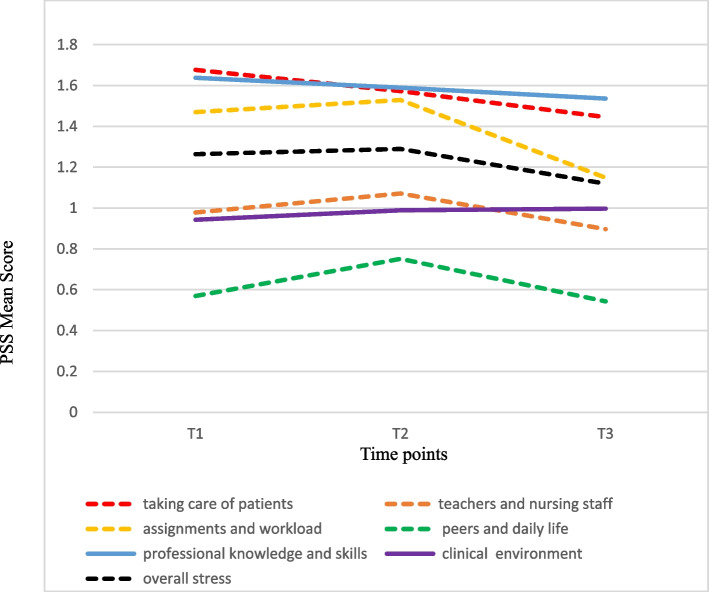
Trajectory of change in Perceived Stress Scale (PSS) among nursing students during one-year clinical practicum

The study results show that the overall perceived stress of students is the highest in T2 and the lowest in T3. Furthermore, the mean score of overall perceived stress in T3 is significantly lower than that in T1 and T2. The mean score in T3 in all PSS subscales of is the lowest, except for the subscale 'clinical environment' (Fig. 1). The mean stress scores in three surveys (T1, T2 and T3) are shown in Table 2.

**Table 2:** Trajectory of change in the PSS, CBI and CCS among nursing students during one-year clinical practicum

item	T1	T2	T3	*Z*	*P*	Post hoc
	Mean (SD)	Mean (SD)	Mean (SD)			
**Perceived Stress**						
Taking care of patients	1.68 (0.67)	1.57 (0.60)	1.45 (0.57)	2.92	0.0035	abc
Teachers and nursing staff	0.98 (0.58)	1.07 (0.56)	0.90 (0.57)	2.21	0.0271	abc
Assignments and workload	1.47 (0.80)	1.53 (0.72)	1.15 (0.71)	0.71	< 0.0001	bc
Peers and daily life	0.57 (0.56)	0.75 (0.62)	0.54 (0.59)	4.35	< 0.0001	ac
Lack of professional knowledge and skills	1.64 (0.69)	1.59 (0.62)	1.54 (0.66)	1.00	NS	-
Clinical environment	0.94 (0.71)	0.99 (0.66)	1.00 (0.70)	0.51	NS	-
Overall stress	1.26 (0.52)	1.29 (0.48)	1.12 (0.50)	0.35	0.0003	bc
**Coping Behaviour**						
Avoidance	0.68 (0.59)	0.88 (0.63)	0.68 (0.58)	4.26	< 0.0001	ac
Problem-solving	2.37 (0.69)	2.23 (0.69)	2.26 (0.65)	2.53	0.0115	a
Stay optimistic	2.68 (0.74)	2.51 (0.75)	2.60 (0.72)	2.56	0.0105	a
Transference	1.80 (0.91)	1.86 (0.86)	2.12 (0.80)	0.81	< 0.0001	bc
**Clinical competence**						
General nursing	3.74 (0.50)	3.69 (0.53)	3.93 (0.51)	1.18	< 0.0001	bc
Cooperation	3.97 (0.59)	3.98 (0.61)	4.03 (0.55)	0.00	NS	-
Management	3.81 (0.60)	3.80 (0.61)	4.03 (0.57)	0.24	< 0.0001	bc
Self-growth	3.90 (0.65)	3.77 (0.62)	3.89 (0.63)	2.04	0.0413	ac
Positiveness	4.02 (0.63)	3.81 (0.68)	3.92 (0.68)	3.56	0.0004	a
Overall clinical competence	3.82 (0.48)	3.75 (0.50)	3.94 (0.49)	1.57	0.0005	bc

*P* value with statistically significant differences (*p* < 0.05) using generalized estimating equation (GEE) for related samples between T1, T2 and T3

NS represents what there is no statistical difference

Post hoc: "a" represents that there is a significant difference between T1 and T2; "b" represents that there is a significant difference between T1 and T3; "c" represents that there is a significant difference between T2 and T3

*PSS* Perceived Stress Scale, *CBI* Coping Behaviour Inventory, *CCS* Clinical Competence Scale

The main source of stress of students in their practicum is 'taking care of patients' (T1) and 'lack of professional knowledge and skills' (T2, T3). The mean scores for 'taking care of patients' in T1, T2 and T3 are 1.68±0.67, 1.57±0.60 and 1.45±0.57, respectively, and there are significant differences among T1, T2 and T3. This finding indicates that students' stress from 'taking care of patients' also decreases over time. However, the mean scores for 'lack of professional knowledge and skills' in T1, T2 and T3 are 1.64±0.69, 1.59±0.62 and 1.54±0.66, respectively, with no significant difference.

### Trajectory of change in coping strategies of nursing students during their practicum

**Fig. 2:**
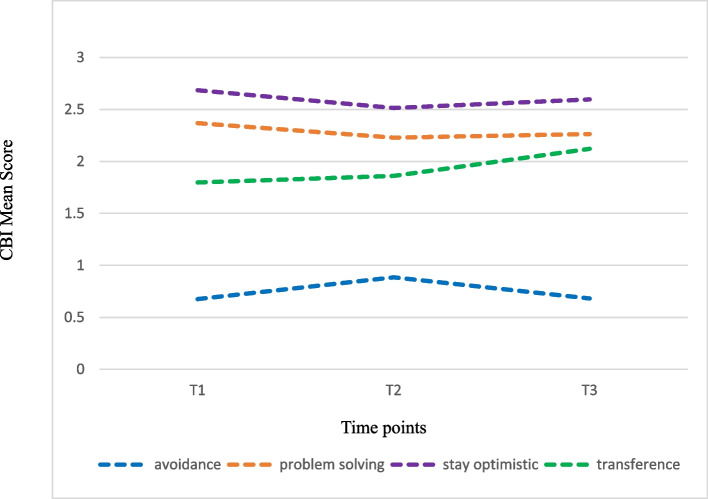
Trajectory of change in Coping Behavior Inventory (CBI) among nursing students during one-year clinical practicum

The four coping strategies of CBI remain the same ranking in three surveys. Students get significantly higher mean scores for 'stay optimistic' and 'problem-solving' in T1 than in T2. It is also found that the mean score for 'avoidance' in T2 (0.88 ± 0.63) is higher than that in T1 (0.68 ± 0.59) and T3 (0.68 ± 0.58), with significant differences. In addition, the mean score for 'transference' in T3 (2.12 ± 0.80) is higher than that in T1 (1.80 ± 0.91) and T2 (1.86 ± 0.86), with significant differences (Table 2, Fig. 2).

### Trajectory of change in clinical competence of nursing students during their practicum

**Fig. 3:**
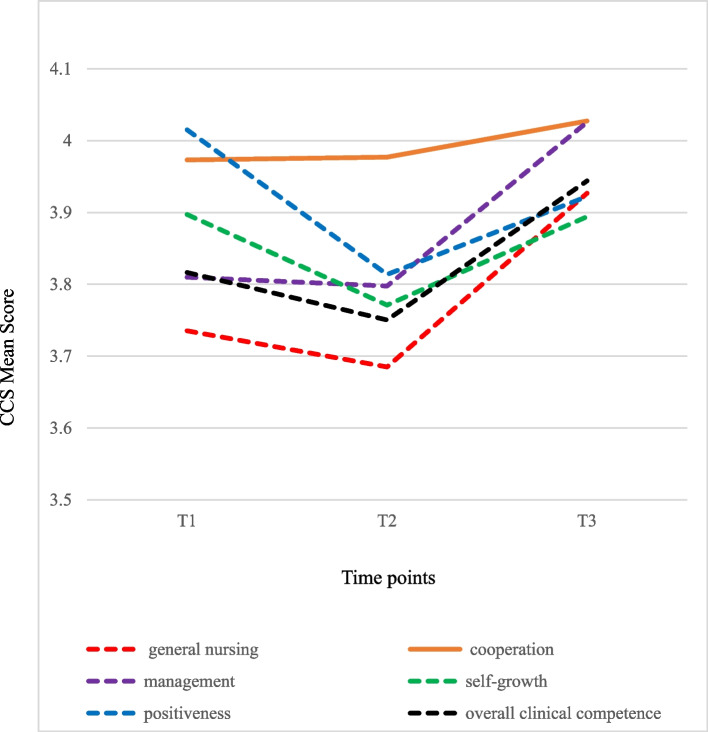
Trajectory of change in Clinical Competence Scale (CCS) among nursing students during one-year clinical practicum

The study results show that the overall clinical competence of students is the highest in T3 and the lowest in T2, and there are significant differences between T3 and T1 and between T3 and T2 but none between T1 and T2. In the CCS subscales, the mean scores of 'general nursing' and 'management' in T3 are higher than those in T1 and T2, with significant differences. The score in T2 is lower than that in T1, but there is no significant difference. Students' mean scores in 'cooperation' increase over time, but there is no significant difference among T1, T2 and T3. Students' scores in 'self-growth' and 'positivity' in T1 are also higher and the lowest in T2, and there are significant differences (Table 2, Fig. 3).

## Discussion

Our study aims to track the trajectory of change in students' stress during a one-year clinical practicum in a long term. This study result shows that the degrees and sources of students' perceived stress during their practicum change. Our study results show that students experience the highest overall perceived stress in T2, followed by that in T1 and the lowest in T3. In addition, the main source of stress in students' practicum is 'taking care of patients' (T1) and 'lack of professional knowledge and skills' (T2, T3).

In recent years, only Bhurtun et al. [21] and Mazalová et al. [19] have tracked the changes of nursing students' stress in their clinical practicum. Mazalová et al. [19] tracked for three consecutive years. Their study results showed that students experience the highest overall perceived stress in the first year, followed by the third year and the lowest in the second year. Furthermore, the main source of students' stress is 'lack of professional knowledge and skills'. Bhurtun et al. [21] tracked for two consecutive years. Their study results showed that students' perceived stress in the second year is higher than that in the first year and that there are significant differences. Similarly, the main source of students' stress is 'lack of professional knowledge and skills'. The collection and tracking time are different, which may affect the result from stress changes. This may be due to our students' experience in the continually one-year practicum, where their experiential learning is not interrupted. Therefore, they are more familiar with the clinical setting. Clinical practicum is essentially experiential learning. It achieves learning through practical operation, observation and reflection. Experience gained by students during the clinical practical training contributes to their better adaptability to real-life conditions [29].

Two things we also found that the degree of stress from 'taking care of patients' decreases gradually over time, and the three surveys are significantly different. In addition, the degree of stress from 'lack of professional knowledge and skills' also decreases gradually over time, though the three surveys are not significantly different. Based on our research results, the possible reasons are inferred as follows. Firstly, students experience more in the following practicum departments (medical-surgical, maternity, paediatric, psychiatric, community health) in T2, which exposes them more frequently to stressful environments. Secondly, when facing a high-level practicum (T2), students must integrate deeper and broader professional knowledge or skills in clinical practice. Thirdly, compared to T3, students must take on more responsibilities in T2, and clinical nursing teachers have stricter requirements for the performance of students' clinical competence as well as the quantity and depth of homework. Therefore, students experience higher overall perceived stress in T2 than in T3. Last but not least, the levels of practicum are increased, but the perceived stress of students from 'taking care of patients' may decrease gradually over time due to the accumulation of experiential learning of continuous practicum time after time.

The coping strategies used by nursing students vary according to the level and source of their stress [22, 23]. Our study results show that the main coping strategy used when students managed stress is 'stay optimistic', followed by it is 'problem-solving'. Our study result is similar to the findings of Mazalová et al. [19], which showed that the main coping strategy students used in the first and second years is 'problem-solving', followed by 'stay optimistic'. The practicum goals of students at different surveying times are not explained in Mazalová et al. [19], but both their and our study show that 'problem-solving' and 'stay optimistic' are the most commonly used coping strategies among nursing student during clinical practicum.

Our study result is different from the findings of Bhurtun et al. [21], which showed that students used all coping strategies more frequently in their second year than in their first year and used 'transference' as their main strategy to cope with stress during their clinical practice. However, nursing students also reported using problem-based coping strategies. Coping is comprised of repeated 'cognitive and behavioural' endeavours undertaken by individuals in order to control extreme stressors whether these are internal and/or external [30]. A recent systematic review has revealed that nursing students tend to use a combination of coping strategies, including effective ones such as problem-solving and stay optimistic, alongside less effective strategies like avoidance and transference [22]. Effective coping strategies encompass actively addressing stress and solving problems and seeking social support to manage negative emotions and seek advice [5, 15, 30]. Less effective strategies like intended to lessen, control, and manage adverse emotional responses by avoidance through self-distraction activities such as watching TV, snacking, and sleeping [5, 21, 23]. It is possible that nursing students use ineffective coping strategies to immediately dampen distress emotions caused by stress in their clinical practicum, after which they are able to identify and use effective coping strategies calmly that were not obvious previously.

In addition, students use 'stay optimistic' and 'problem-solving' more frequently in T1 than in T2 and T3. This result may be due to the fact that, when students participate in clinical practicum for the first time, they provide actual patient care and hands-on training and embody the role of nursing professionals, they would keep optimistic and use problem-solving strategies to manage stress actively. Additionally, students underwent continuous clinical practicum, and they would become familiar with the practicum setting and clinical practice. Therefore, the frequency of using problem-solving and stay optimistic may be decreased in T2 or T3. Alshahrani et al. [30] study showed that nursing students used a range of strategies that had enabled them to positively cope with their first clinical practicum experience. Strategies included use of debriefing sessions with their clinical lecturers and seeking-out their friends and family to talk about their first clinical practicum experiences. Other strategies included being adequately prepared before the clinical practicum, identifying and seeking advice from supportive nursing staff. Lopez et al. [31] study showed that students were stressed while facing challenges head-on during their first clinical practicum. Gradually, students built resilience overtime and were able to adapt to the ward culture through peer support and reframing coping strategies.

It is worth mentioning that our students use more coping strategies on 'avoidance' in T2 than in T1 and T3 and that there are significant differences. At the same time, it is also found that the score of overall perceived stress in T2 is the highest. Does this result indicate that our students tend to adopt avoidance behaviours when facing greater stress? However, the reported relationship between stress levels and the used coping strategies is inconsistent. Al‐Gamal et al. [32] study showed a significantly negative correlation between the total PSS score (stress level) and the use of specific coping strategies, namely problem solving. Two studies [16, 33] reported higher stress levels among students who utilized coping strategies like avoidance or transference strategies. Experiencing a positive clinical learning environment is associated with a lower frequency of using ineffective coping strategies, such as avoidance behaviour [19]. Our study result suggests that we should pay attention to students' perception of stress in their practicum, provide appropriate assistance and guide students to alleviate their stress using problem-focused coping strategies. Problem-focused coping by targeting the root causes of stress is highly suggested [5].

Experiential learning is essential for enhancing the practical and clinical competence development in health-care students because of the intricacy of health-illness phenomena. Experiential learning refers to learning by doing and entails hands on experiences [34]. The experiential learning theory emphasizes the experiential aspect of the learning process; hence, it seeks to continuously change the experiences of the student. This ongoing educational process of alternating the student's experiences help build their knowledge and influence schema [35]. Therefore, nursing schools arrange a series of clinical practicum courses for students so that students can accumulate professional knowledge and skills through repeated experiential learnings. A successful clinical practicum is also important for students' professional development [36]. Good quality for the clinical learning environment has been found to consist of the pedagogical atmosphere on the ward, supervisory relationship, leadership style of the ward manager, and the premises of nursing on the ward [1, 36]. Visiers-Jiménez et al. [1] study showed the correlation between the students' perceptions of their final clinical learning environment and competence was statistically significant and positive. Our research results show that the overall clinical competence, 'general nursing' competence, 'management' competence and 'cooperation' competence of the students are the highest in T3 and the lowest in T2. However, the mean scores for 'self-growth' and 'positivity' are the highest in T1 and the lowest in T2. Based on our research results, the possible reasons are inferred as follows. Firstly, as the practicum level increases, students may accumulate continuous experiential learning time after time to gradually improve their competences in 'general nursing', 'management' and 'cooperation' as well as overall clinical competence. Secondly, students experience different practicum departments (medical-surgical, maternity, paediatric, psychiatric, community health) in T2, and they need to learn different professional knowledge, which may result in students' self-assessed of poor clinical competence. However, in T3, the department is chosen by the students themselves, so students may over-judge their clinical competence. Thirdly, students may perceive greater stress in T2, which lowers their stress adjustment, sense of responsibility and service enthusiasm (positivity competence), thereby reducing their enterprising spirit (self-growth competence).

It is worth mentioning that both the mean scores of overall clinical competence and various subscales are the lowest in T2. In addition, the overall perceived stress in T2 is the highest, and students use the coping strategy 'avoidance' the most frequently. Do these findings indicate that there may be a correlation between the perceived stress, coping strategies and clinical competence of our students during their practicum? This question is worth further analysis in the future. Finally, it is also found that students' self-assessed of overall clinical competence (1–5) T1, T2, and T3 were 3.82, 3.75, and 3.94 respectively, all of which were at a moderate level. Our research result is similar to the findings of [1], which showed that graduating nursing students assessed their overall competence mean on the VAS as 64.5 (0–100), which corresponds to the range for a good level (50–75).

### Strengths and limitations

This study has the following strengths: 1. The study is well organised and applies a longitudinal design, thereby responding to a research gap. 2. Data on the perceived stress, coping strategies and clinical competence of students during clinical practicum are completely collected. This study has the following limitations: 1. The COVID-19 pandemic just occurred during T1 and T2, and so some of the practicums were completed online or in a simulated classroom. Therefore, the scores for perceived stress, coping strategies and clinical competence cannot be used to truly reflect face-to-face and hands-on clinical practicum. 2. The clinical competence scores reported here do not necessarily reflect the actual clinical competence of the students because these were self-reported. 3. We did not fully consider potential factors that may affect perceived stress and coping strategies (such as physical activity, personality traits, interpersonal relationships), which may affect the results of this study. 4. Because there is no control group, it is difficult to know whether the maturation effect during the practicum process and how to affect students' perceived stress and coping strategies. 5. The results of this study cannot be generalized to different regions, universities or professional fields.

## Conclusion

The results show that through experiential learning in clinical practicum at different stages time after time, students' overall perceived stress is the lowest and their overall clinical competence is the highest in the comprehensive clinical nursing practicum (T3). The degree of students' stress from 'taking care of patients' decreases gradually over time. The main coping strategy used when students managed stress is 'stay optimistic'. Furthermore, the results also show that students' overall perceived stress is the highest, the overall clinical competence is the lowest and they used the ‘avoidance’ coping strategy more frequently in advanced training practicum (T2).

Based on our research results, our recommendations are following: 1. Clinical educators should explain the scope and evaluation criteria of clinical practice and the responsibilities of students so that students have practical expectations about the objectives of clinical practicum to avoid unnecessary stress. 2. Nursing educators are encouraged to develop strategies that decrease the level of stress and promote effective coping strategies among nursing students during their clinical practicum. 3. The clinical competence and retention rates of these nursing students in the workplace will still be tracked in the future.

## Data Availability

The datasets used and/or analysed during the current study available from the corresponding author on reasonable request.

## Abbreviations

PSS: Perceived Stress Scale
CBI: Coping Behaviour Inventory
CCS: Clinical Competence Scale
